# Antioxidants, inhibits the growth of foodborne pathogens and reduces nitric oxide activity in LPS-stimulated RAW 264.7 cells of nipa palm vinegar

**DOI:** 10.7717/peerj.12151

**Published:** 2021-09-16

**Authors:** Wilaiwan Senghoi, Wiyada Kwanhian Klangbud

**Affiliations:** 1 Center of Excellence Research for Melioidosis, Walailak University, Thasala, Nakhon Si Thammarat, Thailand; 2 Department of Medical Technology, School of Allied Health Sciences, Walailak University, Thasala, Nakhon Si Thammarat, Thailand

**Keywords:** Nipa palm vinegar, *Nypa fruticans* Wurmb., Antioxidant, Anti-inflammation, Anti-enteropathogenic bacteria

## Abstract

Nipa palm vinegar (NPV) made from the sap of nipa palm (*Nypa fruticans* Wurmb.) has long been used as a local food seasoning and folk medicine. This study compared the bioactive compounds, antioxidant, *in vitro* anti-inflammatory and antimicrobial activities of three NPVs obtained from different plantations based on varied soil and water salinity levels, including fresh water NPV, brackish water NPV and saline water NPV. The analysis results revealed that total phenolic content of saline water NPV had statistically significantly lower than both fresh water and brackish water NPV (*p* < 0.0001). Furthermore percentage of acetic acid in brackish water NPV had statistically significantly lower than both fresh water and saline water. NPV (*p* = 0.002). Nevertheless, total flavonoid and pH, were not significantly different (*p* = 0.144 and 0.066, respectively). The antioxidant activities using three ABTS, DPPH and FRAP methods displayed similar patterns, in which saline water NPV showed the highest antioxidant activities, followed by brackish water and fresh water NPV, respectively. Antimicrobial activity was examined for seven enteropathogenic bacteria. The tested NPVs were found inhibitive against all test cultures with a minimum inhibitory concentration (MIC) of ≤ 7.8 µL/mL. The cytotoxicity of the NPV obtained from different plantations by MTT assay revealed low cytotoxicity. Anti-inflammatory activity was also carried out through the inhibition of nitric oxide production. The fresh water NPV exhibited the highest anti-inflammatory activity with IC50 17.59 ± 0.17 µL/mL, followed by saline and brackish water NPV with IC50 18.12 ± 0.49 and 28.29 ± 2.64 µL/mL, respectively. The findings indicated that NPV from different soil salinities could potentially be natural functional food and developed to antimicrobial and anti-inflammatory medicinal agents with safety.

## Introduction

Nipa palm (*Nypa fruticans* Wurmb) is a palm species found abundant in mangrove forests, particularly in the South, Southeast Asia, and Oceania. Several nipa palm clusters are growing naturally in many areas of the south of Thailand. Nipa palm is locally well known as Ton-Jak. It is a perennial wild plant found naturally growing in estuaries (Prasad et al., 2013). The local people have made many uses of nipa palm, such as utilizing its leaves for making baskets and wrapping cigarettes, its flower stalks for making flyswatter, and its young fruits for making a drink and dessert (Matsui et al., 2014; Udofia & Udo, 2005). Nipa sap is obtained by tapping the fruit stalks (inflorescence). Sugar-rich sap is commonly used for making sweet, beverage, alcohol and vinegar production (Javier & Scott, 2013). The fermented sap is called nipa palm vinegar (NPV). It has been generally used in cooking and associated with various health and medical benefits, including antioxidant, antidiabetic, antilipidemic, hepatoprotective, anti-inflammation and immunomodulation benefits (Beh et al., 2016; Chatatikun & Kwanhian, 2020; Yusoff et al., 2015; Yusoff et al., 2017; Beh et al., 2017; Laklaeng & Kwanhian, 2020). These health benefits may be ascribed to the presence of several bioactive substances in its components, including organic acids, enzymes, amino acids, vitamins and phenolic acids (Myint, 2011; Thanikan, 2018). Nowadays, natural products have become increasingly popular as health-promoting benefits since people are paying more attention to the functional properties of food products (Chen et al., 2016). NPV is conventionally used as a sour food seasoning. Therefore, the ability to inhibit enteric pathogens that may contaminate food is helpful. Different studies have reported that vinegar could be used to inhibit pathogenic bacteria which can contaminate foods, fruits and vegetables, for example, *E. coli * (EHEC) O157:H7, *Shigella sonnei*, *etc.* (Chang & Fang, 2007; Rhee et al., 2003; Sengun & Karapinar, 2004; Wu et al., 2000). However, other critical enteropathogenic bacteria can contaminate food, such as *Vibrio * spp., *Salmonella * spp., *Shigella * spp., *Staphylococcus aureus*,* etc. * (Cheun et al., 2010). The primary substance in vinegar, such as acetic acid and other organic acids, can kill harmful bacteria or inhibit multiplying (Nada, 2013). Nipa palm vinegar has been reported as an effective antimicrobial agent (Myint, 2011). Nipa sap before fermented into NPV was found microbial contaminated in order from most to least amount: lactic acid bacteria, yeasts and coliform (Manel et al., 2011), which were involved in fermented processes. 

Nipa palm can be adapted to muddy soils along with fresh, brackish and saline water, especially in Khanab Nak Sub-district, Pak Phanang District, Nakhon Si Thammarat Province, Thailand. Salinity has been considered a key factor for the existence of nipa palm (Alongi, 2009). The previous study showed that high salinity affected the growth and physiology of nipa palm (Cattarin et al., 2014). Sap production would be controlled by the nipa growth and the amount of Na^+^ in soil by sap producing increases when decreasing the influence of brackish water and the increase of soil organic matter content (Hossain & Islam, 2015). From these data, despite the possibility that the salinity from different planting areas may affect bioactive compounds of NPV. The different bioactive compounds might indicate the difference in their bioactivities and characteristics. However, they are no pharmacological efficacy studies have been performed until now. 

In this respect, it is worth estimating whether the planting areas of nipa palm with different salinity could influence the bioactive compounds, antioxidant, anti-inflammatory and antimicrobial properties of the NPV. Therefore, the main aim of this study was to investigate the nature of bioactive compounds with antioxidant, anti-inflammatory and antimicrobial activities of NPV from different salinity planting areas, including fresh, brackish and saline water in the Khanab Nak sub-district. The findings of this study are expected to develop the Thai NPV to the functional food and promote Thai NPV consumption. Nevertheless, taking a new approach to clarify the difference of NPV from different salinity plantations is of most importance and could help overcome current limitations, with a positive economic impact on the agriculture sector.

## Materials & Methods

### Schematic overview of the experimental program

**Figure 1:**
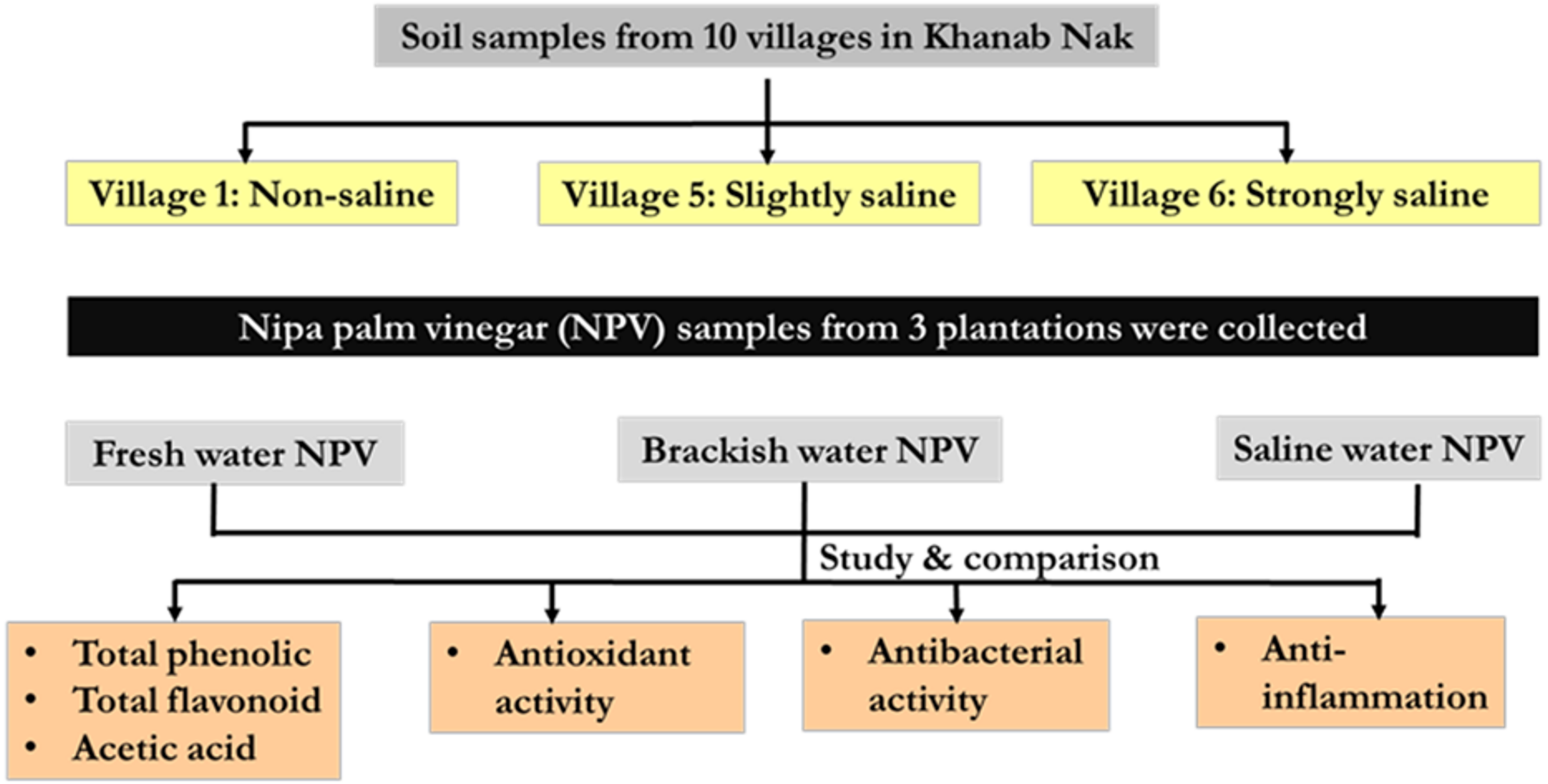
Schematic overview of the experimental program.

Nipa palm vinegar (NPV) samples were from 3 different plantations based on soil salinity (non-saline, slightly saline and strongly saline) of Khanab Nak, Sub-districts of Pak Phanang, Nakhon Si Thammarat, Thailand. NPV samples were determined physiochemical properties and chemical contents, included total phenolic content (TPC), total flavonoid content (TFC), acetic acid content and pH. In addition, they were assessed for antioxidant antibacterial and anti-inflammatory activities, as shown in schematic overview Fig. 1.

### Chemicals and reagents

All chemicals and reagents used were of analytical grade. 2,2′-azinobis-3-ethylbenzothiazoline-6-sulphonic acid (ABTS), 2,2-diphenyl-1-picrylhydrazyl (DPPH), 2, 4, 6-tri (2-pyridyl)-l, 3, 5-triazine (TPTZ), Folin–Ciocalteu phenol reagent, sodium carbonate, gallic acid, quercetin, sodium chloride and aluminum chloride were purchased from Sigma-Aldrich (MO, USA), whereas 6-hydroxy-2,5,7,8-tetramethylchroman-2-carboxylic acid (Trolox) was obtained from Merck (Darmstadt, Germany).

### Determination of soil and water salinity

**Figure 2:**
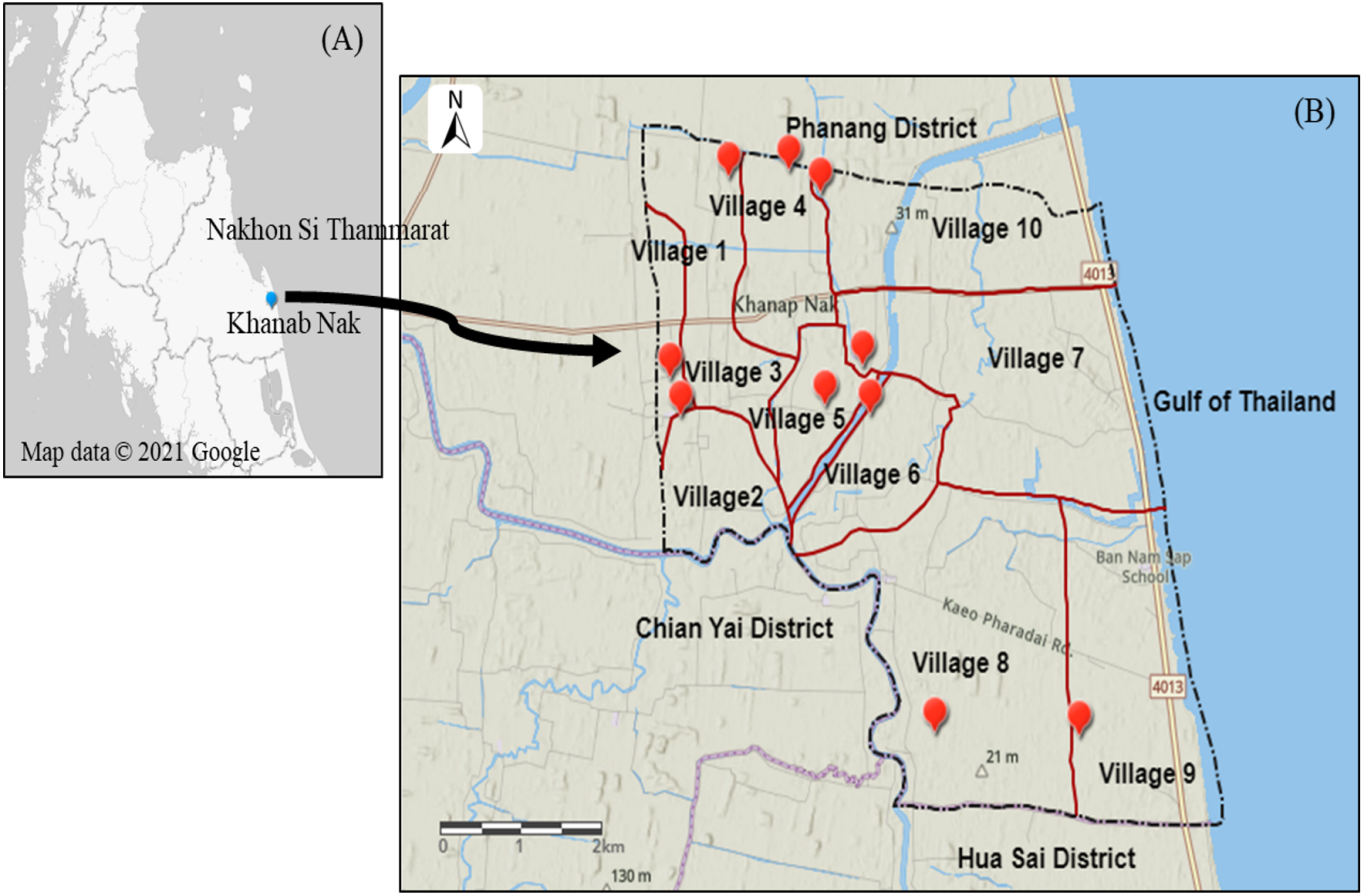
Map of Khanab Nak Sub-district, Pak Phanang District, Nakhon Si Thammarat, Thailand. (A) Location of Khanab Nak Sub-district in Nakhon Si Thammarat. (B) Location of the Khanab Nak area in Pak Phanang District. The map of the study region was created using ArcGIS online.

Khanab Nak, Sub-districts of Pak Phanang, Nakhon Si Thammarat, Thailand, has the potential for Nipa palm plantation (Tripathi & Bencure, 2012). It is divided into 10 villages where are all nipa palm planting areas (Fig. 2). Soil and water samples were collected to study the NPV properties produced from planting areas with different salinities. Soil salinity was analyzed using electrical conductivity (EC) and interpreted according to the laboratory procedures of US Salinity Laboratory Staff (US Salinity Laboratory Staff, 1954). Soil EC was reported in the unit of dS/m. The water samples from three nipa palm planting areas, included non-saline, slightly saline and very strongly saline, were collected and measured. The water salinity was determined by the salinity refractometer (Tran instrument, Petro Centre, Singapore) and was expressed in parts per thousand (ppt) which was interpreted into the degree of salinity.

### Nipa palm vinegar samples

The nipa palm sap was fermented to NPV by the traditional local method (Chatatikun & Kwanhian, 2020). The procedure, in brief, collect nipa palm sap from the cut stems and placed it in a terracotta tank at room temperature for 40 days to allow the natural fermentation process. Nipa palm vinegar has an acidity of 4% to 5%. This study was performed on three NPV samples from different plantations, including villages 1, 5 and 6 of Khanab Nak Sub-district, based on the different soil salinity levels (Fig. 1). All parts of the nipa palm were authenticated and a voucher number (*Nypa fruticans* Wurmb, voucher no. 01518) was deposited at Botanic Garden, Walailak University, Nakhon Si Thammarat, Thailand. Samples were collected from the study areas by cooling transportation. The NPVs were aliquoted and stored in the dark at −20 °C until use.

### Determination of acidity

Colorimetric titration (AOAC, 2000) was used to determine the acetic acid of NPV. A sample and phenolphthalein were titrated with sodium hydroxide. By color changed to red, the titrant volume was used to calculate the percentage of acetic acid concentration in the samples. In addition, pH of NPV samples was also determined by a benchtop pH meter (Mettler-Toledo, LLC, Columbus, OH, USA).

### Determination of total phenolic contents

According to the procedure described in our previous work, the amount of total phenolic in NPV was determined by Folin-Ciocalteu assay (Chatatikun & Kwanhian, 2020). First, 20 µL of samples were mixed with 100 µL of Folin-Ciocalteu reagent and 80 µL of sodium carbonate solution (75 g/L). After the mixture was vortexed for 10 s, it was left for 30 min at room temperature. Absorbance was measured at 765 nm against a blank. Then, the total phenolic content was determined using a calibration curve of gallic acid (0–500 µg/mL) and the results were expressed as microgram gallic acid equivalent per milliliter (µg GEA/mL).

### Determination of total flavonoid contents

The amount of total flavonoid in NPV was investigated using the aluminum chloride colorimetric assay described by Yusoff et al. (2015). First, 50 µL of sample or standard solution was added to 10 µL of 10% aluminum chloride solution, followed by 150 µL of 96% ethanol. Then, 10 µL of 1 M sodium acetate was added to the mixture. All reagents were mixed and incubated for 40 min at room temperature, protected from light. The 96% ethanol was used as a reagent blank. The absorbance was read at 415 nm with a BioTek™ Eon™ microplate spectrophotometer (Thermo Fisher Scientific, Waltham, MA, USA). Quercetin (0–500 µg/mL) was used to make a standard calibration curve. Total flavonoid contents were determined from the standard calibration curve and were expressed as micrograms of quercetin equivalents per milliliter (µg QE/mL).

### Antioxidant property

### ABTS radical scavenging activity

ABTS assay was performed as described by Re et al. (1999). Briefly, to obtain ABTS radical cation (ABTS^+^) solution, 7 mM ABTS and 1 mM potassium persulfate solution were mixed and stored in the dark at room temperature for at least 12 h. Then one mL of ABTS+ solution was diluted with approximately 88 mL of ethanol to obtain an absorbance value of 0.70 ± 0.02 at 734 nm. Afterward, 200 µL of diluted ABTS^+^ solution and 50 µL of each NPV sample at the different concentrations (25, 12.5 6.25, 3.125 and 1.56 µL/mL) or Trolox standard solution were mixed and the mixture was kept in the dark at room temperature for 3 min. Then, the mixture absorbance was measured at 734 nm using the spectrophotometer (Thermo Fisher Scientific, Waltham, MA, USA).

### DPPH radical scavenging activity

The radical scavenging activity of the NPV was measured in DPPH assay according to the method described earlier (Prasad et al., 2011). Briefly, an aliquot of 50 µL of NPV sample (25, 12.5 6.25, 3.125 and 1.56 µL/mL) was mixed with 50 µL of 0.1 mM DPPH radical solution (prepared in ethanol) in a 96-well plate. The reaction mixture was incubated in the dark at room temperature for 30 min. Afterward, the mixture absorbance was read at 515 nm by a microplate reader against a blank (95% ethanol). Vitamin E (0.001-10 mg/mL) was used as positive controls. The percent of radical scavenged was calculated by employing the following equation:

Radical scavenging activity (%) = $ \left( \frac{\mathrm{A~ blank}-\mathrm{A~ sample}}{\mathrm{A~ blank}} \right) \times 100$.

### Ferric reducing antioxidant power (FRAP) assay

The antioxidant capacity of the NPV was estimated according to the previously reported method (Re et al., 1999). Briefly, working FRAP was prepared by mixing 300 mM acetate buffer (pH 3.6): 10 mM TPTZ solution in 40 mM HCl: 20 mM ferric chloride solution, in proportion of 10:1:1 (v/v/v). An aliquot 20 µL of appropriately diluted NPV was mixed with 200 µL of freshly prepared FRAP reagent and mix thoroughly. After 30 min incubation at 37 °C, an intense blue color complex was formed. The absorbance of the reaction mixture was recorded at 593 nm against a blank using a spectrophotometer. The calibration curve was prepared by plotting the absorbance at 593 nm *versus* different concentrations of the standard antioxidant Trolox.

### Determination of antimicrobial activity

Antibacterial activity of the NPV was studied using the broth microdilution method by detecting minimum inhibition concentration (MIC) and minimum bactericidal concentration (MBC) according to the guidelines of Clinical and Laboratory Standard Institute (CLSI, 2019). In this, we mainly focused on the pathogenic enterobacteria associated with food-borne diseases in humans. *Vibrio cholera* [ATCC39541], *Vibrio parahemolyticus* [ATCC17802], *Salmonella enterica* serovar Typhimurium [ATCC14028], *S. paratyphi* A [ATCC9150], *Shigella sonnei* [ATCC25931], *Staphylococcus aureus* [ATCC25923] and *Escherichia coli* [ATCC25922] were used for examining the antibacterial activity of the NPV. Each microorganism was incubated separately with the same volume of three selected NPVs at different concentrations. The final concentrations of the NPVs were serial diluted among 500 to 7.8 µL/mL. The MIC value of the NPV was determined as the lowest concentration that completely inhibited bacterial growth after 48 h of incubation at 37 °C. For the MBC determination, a portion of liquid (5 µL) from each plate well that exhibited no growth was sub-cultured and then, incubated at 37 °C for 24 h. The lowest concentration that revealed no visible bacterial growth after sub-culturing was taken as MBC.

### *In vitro* cytotoxic test

A cell viability test was carried out to evaluate the cytotoxic effects of the NPV on RAW 264.7 cells (ATCC CRL-2278) provided by Dr. Jitbanjong Tangpong. First, the viability was evaluated by using the 3-(4,5-dimethylthiazol-2-yl)-2,5-diphenyltetrazolium bromide (MTT) proliferation assay (Kumar, Nagarajan & Uchil, 2018). Next, the cells were seeded in a 96-well plate at a cell density of 5  ×10^5^ cells/mL with completed Dulbecco’s modified eagle medium (DMEM) and supplemented with 10% fetal bovine serum (FBS) and 2% of 200 × penicillin/streptomycin and 1% of 100 × amphotericin B. Then the plate was incubated at 37 °C, 5% CO_2_ for 24 h. After incubation, the cells were treated with different concentrations of NPV (100, 50, 25, 12.5, 6.25, 3.125, and 1.56 µL/mL) or media alone as negative control and incubated 48 h. Then, 20 µL of MTT solution (5 mg/mL) was added to each well, and the plates were incubated at room temperature for 4 h. Next, a volume of 200 µL of dimethyl sulfoxide (DMSO) was added to each well to dissolve any formazan crystals formed and the absorbance of each well was determined at 570 nm using the spectrophotometer (Thermo Fisher Scientific, Waltham, MA, USA). Finally, the 50% cytotoxicity concentration (CC_50_) of NPV was calculated according to the NPV concentration that affects 50% RAW 264.7 cell death.

### Inhibition of nitric oxide (NO) production

The inhibitory effects of NPV on NO production were evaluated in LPS-activated murine macrophage RAW 264.7 cells, using a method modified from that previously reported (Tham et al., 2010). RAW 264.7 cells were seeded in 96-well culture plates with 5 ×10^5^ cells/mL in DMEM and incubated at 37 °C, 5% CO_2_ for 24 h. The cells were induced with 100 µg/mL LPS in the absence (negative control) or presence of various concentrations of NPV (100, 50, 25, 12.5, 6.25, 3.125, and 1.56 µL/mL). The amount of NO in the cultured medium was measured by the Griess reagent (1% sulfanilamide and 0.1% naphthylethylenediamine in 2.5% phosphoric acid). The absorbance was measured at 550 nm and a reference wavelength of 650 nm. The amount of NO in the sample was calculated from a NO standard curve.

### Statistical analysis

All experiments were performed in triplicates. The data obtained in the study were manifested as mean ± standard deviation (SD). The one-way analysis of variance (ANOVA) was used to examine three performances of different NPV types, while Mann - Whitney U test was used for comparing the mean of two NPV types by using IBM^®^ SPSS^®^ statistics version 23. The statistically significant difference was considered when *p* < 0.05.

## Results

### Soil and water salinity

The lowest EC value was observed in a soil sample in village 1. The EC value in village 1, 2, 3 and 8 was classified as non-saline. The EC value in village 5, 7 and 9 was classified as slightly saline. The soil sample of village 4 and 10 was grouped as moderately saline. The highest EC value was recorded in the soil sample of village 6 (Table 1). Therefore, in this case, three representative varieties of local NPV products were selected from different soil salinity levels, including village1: non-saline, village 5: slightly saline and village 6: strongly saline, as shown in Table 1. The three NPV samples from villages 1, 5 and 6 were defined as fresh water NPV, brackish water NPV and saline water NPV, respectively, according to the salinity level of surface water (Table 2).

**Table 1:** Soil salinity level of nipa palm planting areas from 10 villages in Khanab Nak subdistrict, Pak Phanang district, Nakhon Si Thammarat, Thailand.

**Location in** **Khanab Nak**	**Coordinates**	**EC** **(** **dS** **/** **m** **)**	**Degree of salinity**
Village 1	8°12′53.1″N100°14′00.7″E	0.14	Non-saline
Village 2	8°12′39.9″N100°14′05.2″E	0.15	Non-saline
Village 3	8°14′04.2″N100°14′25.0″E	1.35	Non-saline
Village 4	8°14′06.5″N100°14′49.3″E	5.05	Moderately saline
Village 5	8°12′43.3″N100°15′04.2″E	2.50	Slightly saline
Village 6	8°12′40.3″N100°15′23.0″E	15.63	Strongly saline
Village 7	8°12′57.2″N100°15′19.7″E	3.31	Slightly saline
Village 8	8°10′47.8″N100°15′49.5″E	1.02	Non-saline
Village 9	8°10′46.7″N100°16′49.0″E	3.65	Slightly saline
Village 10	8°13′58.6″N100°15′02.7″E	6.46	Moderately saline

**Table 2:** Water salinity of selected nipa palm planting areas.

**Location in** **Khanab Nak**	**Surface water salinity**	**Degree of water**
Village 1	0.0 ± 0.0 ppt	Fresh water
Village 5	1.0 ± 0.1 ppt	Brackish water
Village 6	9.5 ± 0.1 ppt	Saline water

### Total phenolic, total flavonoid and acetic acid contents

Total phenolic and total flavonoid contents of the NPV samples produced from 3 cultivated areas with different soil and water salinity levels were analyzed. The result showed that the highest total phenolic compound values were found in brackish water NPV (177.16 ± 0.95 µg GAE/mL), followed by fresh water NPV (167.10 ± 10.15 µg GAE/mL) and saline water NPV (80.31 ± 4.00 µg GAE/mL). Meanwhile, the total flavonoid content values ranged from 1.60–2.27 µg QE/mL (Table 3). The NPV with the highest total flavonoid content values were saline water NPV (2.27 ± 0.01 µg QE/mL), followed by fresh water NPV (1.67 ± 0.01 µg QE/mL) and brackish water NPV (1.60 ± 0.01 µg QE/mL).

**Table 3:** Physiochemical properties and chemical contents, included total phenolic content (TPC), total flavonoid content (TFC), acetic acid content and pH of different NPV sources.

**NPV from different planting sources**	**TPC** **(µg GAE/mL)**	**TFC** **(µg QE** **/** **mL** **)**	**%** **Acetic acid**	**pH**
Fresh water NPV	167.10 ± 10.15^a^	1.67 ± 0.01^a^	5.28 ± 0.184^a^	2.79 ± 0.05^a^
Brackish water NPV	177.16 ± 0.95^a^	1.60 ± 0.01^a^	4.07 ± 0.000^b^	2.87 ± 0.20^a^
Saline water NPV	80.31 ± 4.00^b^	2.27 ± 0.01^a^	5.26 ± 0.000^a^	2.80 ± 0.10^a^

**Notes.**

All values show in mean ± SD. ANOVA analysis *p* values of TPC, TFC, % acetic acid and pH were < 0.0001, 0.144, 0.002 and 0.066 respectively. Values in the same column with different superscripts are statistically different (*p* ≤ 0.05).

The acetic acid content plays as the principal quality gauge of vinegar (Sengun, Kilic & Ozturk, 2020). The fresh water NPV exhibited the highest acetic acid content, followed by saline water and brackish water NPV, with the concentration of acetic acid values being 5.28 ± 0.184, 5.26 ± 0.000 and 4.07 ± 0.000%, respectively. While pH values showed a reverse correlation to % acetic acid in order of low to higher pH were fresh water (2.79 ± 0.05), saline water (2.80 ± 0.10) and brackish water NPV (2.87 ± 0.20), respectively (Table 3).

### Antioxidant activity of NPV

**Figure 3:**
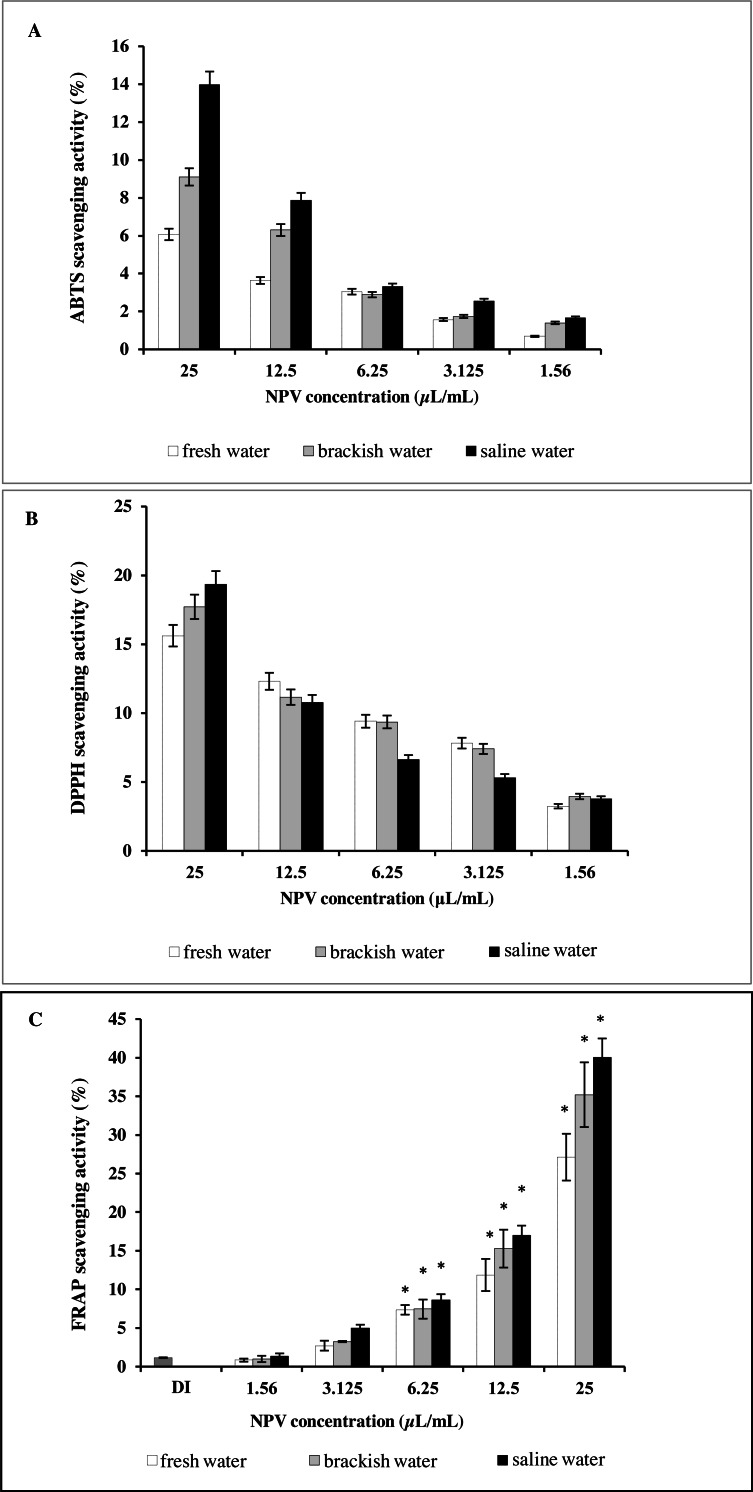
*In vitro* antioxidant assay of fresh water, brackish water and saline water NPV. (A) ABTS radical scavenging activity. (B) DPPH radical scavenging activity and (C) FRAP assay.

Results of antioxidant activity of the NPV samples were compared to each other (Fig. 3). Figure 3A illustrates the effect of three different sources of NPV against ABTS radicals. All the tested samples depicted concentration-dependent radical scavenging activities at a concentration range of 1.56–25 µL/mL. Saline water NPV exhibited the highest scavenging activity of 13.97% at a concentration of 25 µL/mL, followed by brackish water and fresh water NPV, with the 50% scavenging activity (SC50) values being 92.17 ± 1.41, 154.74 ± 19.34 and 265.91 ± 22.81 µL/mL, respectively. Lower SC50 values represented greater scavenging capacities and vice versa.

As shown in Fig. 3B, the highest value obtained with DPPH assay was found in saline water NPV (19.34%), higher than brackish water NPV (17.72%) and fresh water NPV (15.62%) at a concentration of 25 µL/mL. On the other hand, the saline water NPV, at a concentration range of 3.1–12.5 µL/mL, had the lower scavenging activity than brackish water and fresh water NPV, according to the result of SC50 values. SC50 value of saline water NPV (72.53 ± 5.52 µL/mL) was more potent than brackish water (85.29 ±13.28 µL/mL) and fresh water NPV (103.00 ± 22.09 µL/mL). No significant differences were observed between tested samples in the DPPH assay.

Another effective method to evaluate the antioxidant activity of plant polyphenol is FRAP. Like other antioxidant assays, saline water NPV showed a high reducing power (40.05%) followed by brackish water (35.20%) and fresh water NPV (27.13%) at 25 µL/mL (Fig. 3C). All the samples displayed a concentration-dependent activity.

### Anti-enteropathogenic bacteria activity

The MIC and MBC of the vinegar samples were assessed against seven pathogens. The study results exhibited that three different sources of the NPV have similar MIC but different MBC values (Table 4). In all cases, the similarity of MIC values was ≤ 7.8 µL/mL among the strains of the seven bacterial species, displaying intraspecies homogeneous susceptibility. Moreover, the highest growth-inhibitory and bactericidal effects were observed in brackish water and saline water NPV against *V. cholera*, *V. parahaemolyticus*, *S. aureus* and *E. coli* with MIC and MBC of ≤ 7.8 µL/mL. In contrast, fresh water NPV showed the highest bactericidal effect against *V. cholera*, *V. parahemolyticus* and *S. sonnei*. These results could suggest that all three vinegar samples from different soil salinity were effective against both gram-positive and gram-negative bacteria, enteropathogenic bacteria.

**Table 4:** The minimum inhibition concentration (MIC) and minimum bactericidal concentration (MBC) values of NPVs from different sources (Concentration unit = µL/mL).

**Enteropathogenic Bacteria**	**Fresh water** **NPV**		**Brackish water** **NPV**		**Saline water** **NPV**	
	**MIC**	**MBC**	**MIC**	**MBC**	**MIC**	**MBC**
*V.cholera*	≤7.8	≤7.8	≤7.8	≤7.8	≤7.8	≤7.8
*V.parahemolyticus*	≤7.8	≤7.8	≤7.8	≤7.8	≤7.8	≤7.8
*S.typhimurium*	≤7.8	31.2	≤7.8	31.2	≤7.8	31.2
*S.paratyphi*	≤7.8	15.6	≤7.8	31.2	≤7.8	31.2
*S.sonnei*	≤7.8	≤7.8	≤7.8	31.2	≤7.8	62.5
*S.aureus*	≤7.8	15.6	≤7.8	≤7.8	≤7.8	≤7.8
*E.coli*	≤7.8	15.6	≤7.8	≤7.8	≤7.8	≤7.8

### The cytotoxicity effect of the NPV on RAW 264.7 cells

**Figure 4:**
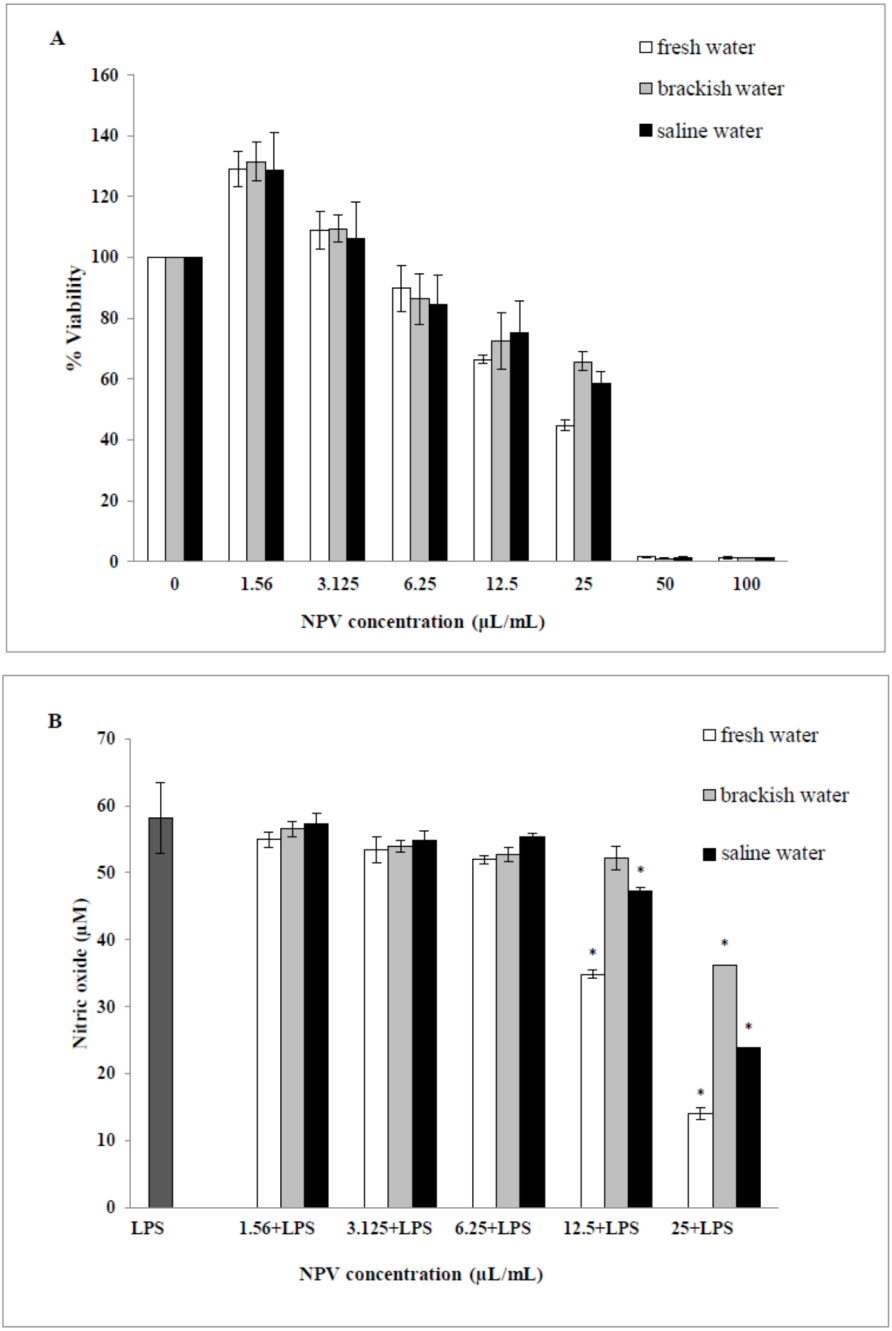
Effects of fresh water, brackish water and saline water NPV on cell viability and NO production in LPS-induced RAW 264.7 mouse macrophages. (A) Cell viability was evaluated using a MTT assay and (B) NO production was measured by the Griess reaction. Values are the mean ± standard deviation of three independent experiments. An asterisk (*) indicates a significance at the level of *p* < 0.05 compare to LPS treated group only.

The cytotoxic effects of the NPV samples on the viability of the RAW 264.7 cells were performed. Values obtained from MTT assays of fresh water, brackish water and saline water NPV were presented as percent cell viability in Fig. 4A. From the result, percent viability values of cells were revealed to diminish with increased concentration (6.25–100 µL/mL). On the other hand, the lower concentration of three NPV samples (1.56 and 3.125 µL/mL) could enhance cell viability. The fresh water NPV had the highest cytotoxicity on RAW 264.7 cells, with a CC50 value of 39.56 ± 2.16 µL/mL, followed by the saline water NPV with a CC50 value of 39.86 ± 5.34 µL/mL, and the brackish water NPV showed the lowest cytotoxicity with a CC50 value of 42.05 ± 3.68 µL/mL (Table 5). The differences were not significant. NPVs at concentrations ranging from 1.56–100 µL/mL did not cause any cytotoxic effect to RAW 264.7 cells, suggesting the effect of NPVs on inhibition of NO production was not due to its cytotoxicity.

### Inhibition of nitric oxide production

The ability of the NPV produced from different sources in reducing the inflammation in LPS-activated RAW 264.7 macrophages was investigated through the ability of the samples to decrease the level of NO. As shown in Fig. 4B, fresh water and saline water-treated cells could reduce the NO level significantly at a concentration of 12.5 and 25 µL/mL in a dose-dependent manner (*p < 0.05*). However, the degree of NO production was remarkably decreased in fresh water NPV-treated cells more than in saline water NPV-treated cells. In contrast, brackish water NPV had a significant inhibitory effect on LPS-induced NO production at 25 µL/mL (*p < 0.05*), but not at lower concentrations of 1.56 to 12.5 µL/mL. The anti-inflammatory effects of the treatment were also exhibited by IC50 (50% inhibitory concentration) values in which fresh water NPV showed the lowest IC50 values followed by saline water and brackish water NPV, with the IC50 values being 17.59 ± 0.17, 18.12 ± 0.49 and 28.29 ± 2.64 µL/mL, respectively (Table 5). The lower IC50 value, the more influential the anti-NO production potential of NPVs.

## Discussion

This study was conducted to elucidate the influence of plantation salinity of nipa palm (*Nypa fruticans* Wurmb) on the bioactive compounds and biological activity of nipa palm vinegar. As such, we analyzed the total phenolic and flavonoid contents, antioxidant, antibacterial and anti-inflammatory properties of traditional nipa palm vinegar, produced in Khanab Nak Sub-district, Thailand.

The raw materials and fermentation processes affect vinegar’s total phenolic and flavonoid contents (Davalos, Bartolome & Gomez-Cordoves, 2005). In addition, substrate selection for vinegar production is an important factor in considering the final phenolic content (Kelebek et al., 2017). The acidity of different fruit vinegar ranged from 1.11–5.61% acetic acid (Sengun, Kilic & Ozturk, 2020). Similarly, the traditional and industrial vinegar samples varied from 0.32 to 7.20% acetic acid (Ozturk et al., 2015). In the previous research, the acetic acid content of both nipa palm vinegar samples produced from two provinces in the south of Thailand contained about 5–6% (Wonghat et al., 2016). From the data presented in Table 3, it could be concluded that different salinity levels in soil and water where nipa palm thrives might influence the bioactive components, especially the total phenolic content of nipa palm vinegar. However, the other factors can influence nipa palm growing and sap properties, including rainfall, fertility, phosphorous content etc. (Cheablam & Chanklap, 2020).

**Table 5:** Cytotoxicity and anti-nitric oxide (NO) production capacity of fresh water, brackish water and saline water NPV.

**NPV from different planting sources**	**Cytotoxicity** **CC**_**50**_ **(** **µL** **/** **mL** **)**	**Anti** **-** **NO production** **IC**_**50**_ **(** **µL** **/** **mL** **)**
Fresh water NPV	39.56 ± 2.16	17.59 ± 0.17
Brackish water NPV	42.05 ± 3.68	28.29 ± 2.64
Saline water NPV	39.86 ± 5.34	18.12 ± 0.49

Phenolic acids in nipa palm vinegar can scavenge superoxide anion and free radicals both *in vitro* and *in vivo* resulting in a potent antioxidant activity (Beh et al., 2016; Yusoff et al., 2015). In order to determine the present antioxidant compounds in the NPV obtained from different sources, the antioxidant activity of fresh water NPV, brackish water NPV and saline water NPV was assessed using three methods of ABTS, DPPH radical-scavenging and FRAP assay. All three *in vitro* antioxidant assays revealed similar antioxidant patterns of the samples by which the saline water NPV showed the most potent antioxidant activity. A distinctive trend was examined whereby the antioxidant activity of the saline water NPV exceeded that of brackish water NPV, which was in order superior to that of the fresh water NPV. Previous reports suggested that total phenolics in vinegar majorly contribute to antioxidant activities (Yusoff et al., 2015). Although saline water NPV exhibited the lowest total phenolic contents, it exerted the most potent antioxidant capacities. The findings in the present study were consistent with previous reports, which showed that antioxidant activity does not correlate with high amounts of phenolics (Kähkönen et al., 1999). It was noted that some chemical groups of sugars or organic acids might be present in the vinegar that might also react with Folin-Ciocalteu reagent and interfere with the result (Kähkönen et al., 1999; Li et al., 2018). Nevertheless, the differences in antioxidant activity among vinegar were derived from their different phenolic compounds and other contents showing antioxidant activities in the samples (Davalos, Bartolome & Gomez-Cordoves, 2005). In vinegar, the content of organic acids and polyphenols is variable and depends on several factors, mainly raw materials, processing techniques, and microbiological growth (Ren et al., 2016).

As shown in Table 4, the NPV from different planting areas showed strong antimicrobial activity against several enteric pathogens. The previous study has shown that the antimicrobial activity of nipa palm vinegar was found to be more potent than that of fresh sap (Myint, 2011). The findings agreed with earlier reports that revealed that fruit vinegar showed antimicrobial activity against nine pathogens associated with food-borne diseases (Wonghat et al., 2016). Similar results were also found out. Furthermore, they researched fig and mulberry vinegar regarding antimicrobial activity against *Bacillus subtilis*, *Enterococcus faecalis*, *Listeria monocytogenes*, *Pediococcus acidilactici*, *S. Typhimurium*, *S. aureus*, *E. coli* and *E. coli* O157:H7. The potential of home-made vinegar as antimicrobial substances could partly be involved with their total phenolic and acid contents (Yücelşengün & Kıç, 2020). Antimicrobial effect of vinegar followed upon polyphenols. These compounds combine with the peptidoglycan and phospholipid bilayer of the outer membrane of bacteria to destroy the integrity of the cell membrane and interfere with the activities of enzymes present in bacteria (Gradisar et al., 2007; Sirk et al., 2008; Taguri, Tanaka & Kouno, 2006; Yoda et al., 2004). However, the present findings found that the phenolic and acid contents contained in the NPV may not contribute significantly to the antimicrobial activity of the NPV. Although several studies investigated the antimicrobial effect of traditionally produced vinegar, no information was found in the literature on comparing local nipa palm vinegar produced from different plantation salinity in the South of Thailand.

NO inhibitors represent an important therapeutic advance in managing inflammatory diseases (Sharma, Al-Omran & Parvathy, 2007). The results indicated that a high concentration of three vinegar samples from different sources might be involved in anti-inflammatory activities by inhibiting NO production. This finding was consistent with the previous report, which indicated that a high dose of nipa palm vinegar effectively abated lipid deposition and suppressed the expression of the inflammatory marker NF-κB and iNOS, resulting in lower levels of NO in obese mice (Lovly & Merlee Teresa, 2018). The effectiveness of nipa palm vinegar in anti-inflammatory activity was also reported by *in vivo* studies. It has been shown that phenolic acids present in nipa palm vinegar could improve oxidative stress, inflammation and liver damage of nipa vinegar-treated obese mice (Beh et al., 2016). To the best of our knowledge, this study is the first to examine the anti-inflammatory effects of NPV on LPS-stimulated RAW 264.7 cells. It was surprising that brackish water NPV containing higher phenolic contents than fresh water and saline water NPV showed the lowest anti-inflammatory activity to RAW 264.7 cells. It was implied that NPV might include non-phenolic compounds with anti-inflammatory properties, which is the highest activity exhibited in the fresh water NPV. However, it also exhibited high cytotoxicity to RAW 264.7 cells at 25 µL/mL. On that point, it was not definite whether the anti-inflammatory activity resulted from cell death or the veritable inhibitory effect of the compounds present in the vinegar samples, or whether the vinegar samples possessed compounds with cytotoxicity and anti-inflammatory activities. The primary anti-inflammatory compound in the NPV needs to be further studied. Further studies are important to determine the precise mechanisms involved fully.

The present study results indicated NPV, sourced from Khanab Nak Sub-district, Pak Phanang District, Nakhon Si Thammarat Province, Thailand, are suitable for food seasoning and alternative medicine. For example, food seasoned by NPV can prevent diarrhea by killing the contaminated enteropathogenic bacteria. Moreover, antioxidant and anti-inflammation capacities of NPV can be used as an alternative medicine to protect or treat the diseases that pathogenesis caused by inflammation and oxidative stress, for example, gastrointestinal mucosal diseases (Bhattacharyya et al., 2014), neurodegenerative diseases (Fischer & Maier, 2015), *etc.*

## Conclusions

It has been indicated that the amount of soil and water salinity levels significantly affect the total phenolic and total flavonoid contents of NPVs. The antioxidant and anti-inflammatory activities of NPVs showed non-significant correlations with their amount of total phenolic and flavonoid compounds. However, all of the different NPVs exerted their antioxidant, anti-enteropathogenic bacteria, and anti-inflammation activities. These findings imply that NPV has significant potential to be used as functional food and folk medicine due to its antioxidant, anti-enteropathogenic bacteria, and anti-inflammation activities.

## Supplemental Information

10.7717/peerj.12151/supp-1 Supplemental Information 1 Raw data

## Additional Information and Declarations

### Data Availability

The following information was supplied regarding data availability: The raw measurements are available in a Supplementary File.
